# Dynamic Histone H3 Modifications Regulate Meiosis Initiation via Respiration

**DOI:** 10.3389/fcell.2021.646214

**Published:** 2021-04-01

**Authors:** Jian Shi, Yanjie Ma, Hui Hua, Yujiao Liu, Wei Li, Hongxiu Yu, Chao Liu

**Affiliations:** ^1^State Key Laboratory of Stem Cell and Reproductive Biology, Institute of Zoology, Chinese Academy of Sciences, College of Life Sciences, University of Chinese Academy of Sciences, Stem Cell and Regenerative Medicine Innovation Institute, Chinese Academy of Sciences, Beijing, China; ^2^State Key Laboratory of Proteomics, National Center for Protein Sciences, Beijing Institute of Lifeomics, Beijing, China; ^3^Shanghai Stomatological Hospital & Institutes of Biomedical Sciences, Fudan University, Shanghai, China

**Keywords:** histone modifications, H3K18 acetylation, meiosis initiation, *RIM101*, *SMP1*

## Abstract

Meiosis is essential for genetic stability and diversity during sexual reproduction in most eukaryotes. Chromatin structure and gene expression are drastically changed during meiosis, and various histone modifications have been reported to participate in this unique process. However, the dynamic of histone modifications during meiosis is still not well investigated. Here, by using multiple reaction monitoring (MRM) based LC-MS/MS, we detected dynamic changes of histone H3 lysine post-translational modifications (PTMs). We firstly quantified the precise percentage of H3 modifications on different lysine sites during mouse and yeast meiosis, and found H3 acetylation and methylation were dramatically changed. To further study the potential functions of H3 acetylation and methylation in meiosis, we performed histone H3 lysine mutant screening in yeast, and found that yeast strains lacking H3K18 acetylation (H3K18ac) failed to initiate meiosis due to insufficient *IME1* expression. Further studies showed that the absence of H3K18ac impaired respiration, leading to the reduction of Rim101p, which further upregulated a negative regulator of *IME1* transcription, Smp1p. Together, our studies reveal a novel meiosis initiation pathway mediated by histone H3 modifications.

## Introduction

To retain genetic stability and variety, most higher eukaryotes produce offspring through sexual reproduction, which includes the production and fusion of two haploid gametes (Smith et al., 2008; Fu et al., 2019). The production of haploid gamete is termed as gametogenesis, in which the diploid precursor germ cells halve the genome content to haploid through meiosis and subsequently develop into fertilization-competent gametes (Lesch and Page, 2012; Kracklauer et al., 2013). In some multicellular organisms, two types of gametes are produced through distinct differentiation processes, including oogenesis and spermatogenesis (Kracklauer et al., 2013; Sacks et al., 2018). Spermatogenesis is a continuous process that includes spermatogonial mitosis, spermatocytic meiosis and spermiogenesis (Roosen-Runge, 1962; Hess and Renato de Franca, 2008), whereas the differentiation of female germ cells is frequently arrested during oocyte maturation (Von Stetina and Orr-Weaver, 2011). Although the development of gametes often display some specialized differentiation processes (Lesch and Page, 2012), the key events of gametogenesis, especially for meiosis, are relatively conserved from single cell organism yeast to multicellular organisms such as worms, flies and mice (Bolcun-Filas and Schimenti, 2012). As a widely used model organism, yeast plays crucial role in understanding mammalian gametogenesis and offers valuable knowledge of meiosis, such as meiotic DNA double-strand breaks (DSBs) formation, meiotic recombination, and synapsis. Many mammalian meiotic key regulators are identified based on the conserved domains in yeast (Bolcun-Filas and Schimenti, 2012). Yeast sporulation is similar to spermatogenesis to some extent. The early phase of yeast sporulation, meiosis itself and spore formation stages roughly correspond to the spermatogonia, spermatocytes and round spermatid stages during spermatogenesis in mouse (Cooper and Strich, 2011). Therefore, these two models build the way to understand the molecular mechanism underlying gametogenesis regulation.

Meiosis is an integral part of gametogenesis (Sacks et al., 2018). During meiosis, chromatin structure is dynamically changed, and a series of events takes place to ensure genetic stability and diversity, such as the programmed DSBs formation, meiotic recombination, homologous chromosomes synapsis and crossover formation (Zickler and Kleckner, 2015; Hillers et al., 2017). A number of mechanisms are used to ensure proper chromatin organization, including chromatin remodelers, non-coding regulatory RNAs (ncRNA), histone variants and histone modifications (Crespo et al., 2020). Any defects in these events may cause genome instability, which is associated with the failure of haploid gamete production and infertility (Wang et al., 2017, 2019).

Post-transcriptional modifications (PTMs) of histones are essential for regulating chromatin structure and gene expression, and extensively involved in many vital cellular processes (Bannister and Kouzarides, 2011; Tessarz and Kouzarides, 2014). Until now, various histone modifications, such as acetylation, methylation, phosphorylation and ubiquitination, have been reported to participate in meiosis (Grunstein, 1997; Tan et al., 2011; Zentner and Henikoff, 2013). These histone modifications could work individually or comprehensively as a “histone code” to regulate meiosis (Jenuwein and Allis, 2001; Koprinarova et al., 2016). For example, H2A.X phosphorylation on serine 139 (gamma H2A.X) could facilitate meiotic DSB repair and is essential for meiotic sex chromosome inactivation (MSCI) (Fernandez-Capetillo et al., 2003). H2BK120 mono-ubiquitination regulates meiotic recombination by promoting chromatin relaxation (Xu et al., 2016). H3K4 trimethylation is enriched at meiotic DSB sites to initiate meiotic recombination (Acquaviva et al., 2013). H4K44 acetylation could promote chromatin accessibility to facilitate meiotic recombination (Hu et al., 2015). Although a number of studies have been performed to decipher histone codes used during meiosis, the dynamics of histone modifications and their functional roles in meiosis are still not well investigated.

Here, we performed multiple reaction monitoring (MRM) based LC-MS/MS to examine the dynamic changes of histone H3 lysine post-translational modifications during meiosis in both mice and yeast. We found H3 acetylation and methylation were dramatically changed during meiosis. To further study their potential functions, we performed a systematic mutational screen on H3 lysine in yeast and found that H3K18 acetylation (H3K18ac) was critical for meiosis initiation. Further studies showed the absence of H3K18ac influenced yeast respiration, which further downregulated the expression of Rim101p. In addition, deficiency of Rim101p activated the expression of *SMP1*, an inhibitor of *IME1* transcription, which finally led to the failure of meiosis initiation. Therefore, our studies uncover a new relationship between histone H3 modifications and meiosis initiation.

## Materials and Methods

### Strains and Plasmids

All strains and plasmids used in this study were described in Supplementary Tables 1, 2, respectively. Histone shuffling strains were constructed using similar methods described in a previous study (Govin et al., 2010). Wild-type (WT) histone H3 and H4 were provided by *HHT1-HHF1* in plasmid pYES2 and then this plasmid was replaced with the pRS303 carrying mutant *HHT1-HHF1.* All deletion strains were homozygous, generated using a PCR-mediated gene replacement method as previously described (Govin et al., 2010). Overexpression plasmids were created by inserting genes into a pYC2-*CUP1* carrier.

### Preparation of Standard Isotope-Labeled Histone Peptides

Standard isotope-labeled histone peptides (Supplementary Tables 3, 5) were prepared as previously described (Gao et al., 2014). Briefly, stock solutions of isotope-labeled histone peptides were prepared at concentrations of 100 μM in 30% acetonitrile. The stock solutions were further diluted by sterile water to get a succession of working standards. Then moderate amounts of standard solution were mixed with 20 μL testing samples to final concentrations of 1, 5, 10, 50, 100, 500, and 1,000 nM as calibration standards. The same methods were used for quality control samples preparation, and the low, medium, and high concentrations were 3, 100, and 850 nM, respectively. All labeled-peptides were mixed at the final concentration of 100 nM in 30% acetonitrile. All prepared solutions and samples were kept at −80°C.

### Histone Extraction and Purification

Samples for MRM-based LC-MS/MS were collected from mouse [spermatogonia (*n* = 4), spermatocytes (*n* = 4) and round spermatids (*n* = 3)] and yeast [incubating with sporulation medium at 0 h (*n* = 2), 4 h (*n* = 3), and 12 h (*n* = 3)]. Histone proteins were extracted and depurated as previously described (Shechter et al., 2007). Briefly, about 5 × 10^6^ cells were pyrolyzed in 500 μL hypotonic lysis buffer (10 mM Tris-HCl pH 8.0, 1 mM KCl, 1.5 mM MgCl_2_, 1 mM DTT, and cocktail of protease inhibitors) at 4°C for 40 min. Samples were then centrifuged for 10 min at 10,000 × *g* to deposit nuclei. Nuclei were digested with 0.4 M H_2_SO_4_, deposited with 100% (w/v) trichloroacetic acid (TCA), and washed twice with ice-cold acetone to retrieve core histones. Histone proteins were further dephlegmated on a C8 column (150 mm × 4.6 mm, Agilent), using an Agilent series 1200 system (Waldbronn, Germany). Finally, histone H3 fractions were mixed together and exsiccated using a SpeedVac for further experiments.

### Histones Derivatization and Digestion

The histone N-propionylation derivatization was performed as previously described (Liao et al., 2013; Gao et al., 2014). The extracted histone H3 fraction pool was redissolved in 100 μL sterile water, and 10 μL histone H3 solution was derivatized in the PD buffer (100 mM NHS acid, 25 mM NH_4_HCO_3_ and 50% acetonitrile) for 30 min at 50°C. Next, the histone H3 were concentrated to dryness with a SpeedVac and digested with trypsin in 25 mM NH_4_HCO_3_ overnight. Finally, the digested peptides were concentrated to dryness again and derivatized in the PD buffer. Before LC-MS analysis, the derivatized peptides were redissolved in 50 μL sterile water and then combined with 50 μL isotope labeled peptides mixture. 5 μL of the final solution was loaded onto the LC-MS/MS system for further analysis.

### LC-MS/MS Analysis

Similar instruments and methods were used as previously described (Gao et al., 2014). Samples were analyzed using a configuration of high-performance liquid chromatography with a Shimadzu Prominence UFLC system (Shimadzu Scientific Instruments, Pleasanton, CA, United States), electrospray Ionization (ESI) with a TurboIonspray probe (AB Sciex, United States) and a QTRAP 5500 mass spectrometer (AB Sciex, United States). An Agilent Zorbax 300 SB-C18 column (150 mm × 4.6 mm, 5 μm; Agilent, United States) was used for chromatographic separation with 0.1% formic acid in sterile water as solvent A and 0.1% formic acid in acetonitrile (v/v) as solvent B. Samples were gradient eluted by a series of solvent: 2% B for 0-2.0 min; 2-30% B for 2.0-30 min; 30-90% B for 30-31 min, 90% B for 31-34 min, and were finally equilibrated to 2% B at a flow rate of 0.6 mL/min for 4 min. The infusion sample volume was 5 μL, the temperature of column was held at 25°C. The voltage of ion spray was set to 5000 V, and the temperature of the turbo spray was 550°C. 60 and 65 arbitrary units was set for nebulizer gas and heater gas, respectively. The curtain gas was set at 40 arbitrary units and turned on the interface heater. The collision energy and declustering potential for all isotope-labeled and endogenous peptides were adjusted to attain maximum MRM intensity. According to their retention times on the HPLC column, the isotope-labeled and endogenous histone peptides were separated in mass spectrometry. Dwell times for each MRM transition were 0.02 s. The AB Sciex analyst software 1.6.1 was used to control and synchronize all the data.

### Quantification of Histone Modifications

The quantification of the histone modifications was performed as previously described (Gao et al., 2014). The concentration (C) of isotope labeled synthetic histone modification peptides were used as a standard, peak area _peptide_ and peak area _labeled peptides_ were achieved using LC-MS/MS analysis. The targeted endosome histone modification peptide concentration was calculated as below: C _peptide_ = C _labeled peptide_ × [peak area _peptide_/peak area _labeled peptide]_. To achieve the relative quantitation of a specific kind of modification on the lysine site in H3, the concentration of the targeted modification divided by the concentrations of all peptides on this targeted site. Using H3K4me1 as an example, the amount of H3K4me1 was calculated as below: Percentage _H__3__K__4__me__1_ = Σ H3K4me1_containing peptides_/[Σ H3K4_containing peptides_ + Σ H3K4me1_containing peptides_ + Σ H3K4me2_containing peptides_ + Σ H3K4me3_containing peptides_] × 100%. As some peptides were isobaric and difficult to be resolved by HPLC or precursor peptide ions, the b^3+^ fragment ions were selected in the MRM transitions to distinguish these peptides.

### Isolation of Mouse Spermatogenic Cells

Testes from 7-day, 17-day, and 8-week old C57BL/6 mice were used to obtain spermatogonia, spermatocytes and round spermatids, respectively, following a method previously described (Xu et al., 2016). Briefly, testes were obtained and decapsulated. Seminiferous tubules were laniated with tweezers into small pieces and suspended by 8 ml PBS with 1 mg/ml hyaluronidase (Sigma, H3506, St. Louis, MO, United States) and 1 mg/ml collagenase (Sigma, C5138, St. Louis, MO, United States). Then, the samples were gently shaken in 37°C water bath for 5 min. After pipetting, the diffused seminiferous tubules and cells were kept at 37°C for another 5 min shaking softly. Then, the samples were centrifuged at 200 × *g* for 5 min at 4°C. After washed once by PBS, the sediments were re-suspended in 15 ml of PBS with 1 mg/ml DNase I and 0.25% Trypsin. Thereafter, the samples were shaking gently in 37°C water bath for 5 min. Then, the cells were assembled and washed by PBS with 0.5% BSA before filtrated through a 40 μm Nylon Cell Strainer. The cells were carefully loaded on a 2–4% BSA gradient in PBS and separated through sedimentation by gravity. The separate cells were collected into different tubes. The cells from each tube were examined with light microscopy to confirm cell-type and purity. The samples containing same cell type (spermatogonia, spermatocytes and round spermatids) with proper purity (≥90%) were collected together.

### Sporulation Conditions

After growth in YPD medium (1% yeast extract, 2% peptone, and 2% glucose) or SD medium (synthetic complete medium with glucose without the corresponding essential amino acid) for 24 h, yeast was deliquesced with YPA medium (1% yeast extract, 2% peptone, and 2% potassium acetate) to OD_600_ = 0.3. Following growth for 10 h at 30°C, cells were gathered, washed, and resuspended in SPM (a sporulation medium, 2% potassium acetate) to OD_600_ = 1.9 and cultured at 30°C for sporulation. In addition, another method was used for WT and respiratory defective cells for sporulation, which was induced by rapamycin as previously depicted (Zheng and Schreiber, 1997). The strains were cultivated in YPD for 24 h shaking vigorously. When cells reached the G1 phase, they were aliquoted into two aliquots. One aliquot was cultivated with methanol as a control, and the other aliquot was treated with rapamycin at a final concentration of 100 nM. To evaluate the sporulation efficiency, meiotic nuclear divisions were used as a surrogate and observed by treating DNA with 1 mg/mL DAPI (4′, 6-diamidino-2-phenylindole). The cells were gathered at the designated times, and then fixed with 1 mL 70% ethanol for subsequent DAPI treating. A Nikon Eclipse Ti microscope (Eclipse Ti-S; Nikon, Tokyo, Japan) was used to obtain and analysis the images.

### Flow Cytometry

About 1 × 10^7^ cells were fixed overnight with 1 mL cold 70% ethanol, and then resuspended in 1 mL 50 mM sodium citrate for DNA content analysis. The cells were centrifuged at 845 × *g* for 5 min, and the sediments were digested with RNase A (Sigma Aldrich, Shanghai, China) for 2 h at 37°C. After sonication for 2 s at 20% power, the samples were stained with 1 mM Sytox Green (Molecular Probes, Eugene, OR, United States). A BD FACS vantage SE Flow Cytometry System (BD, Franklin Lakes, NJ, United States) was used to analysis the results.

### Yeast Growth Sensitivity

Yeast strains were cultured in YPD or SD medium to OD_600_ of 1.0 at 30°C. The cells were gathered, washed, and serially diluted. Each dilution was then spotted onto an auxotrophic plate containing glucose or glycerol and then cultured for 2 days at 30°C.

### Immunoblotting

After mild alkaline treatment, cells were boiled in a standard electrophoresis loading buffer similar as previously described (Kushnirov, 2000). The protein samples were split by SDS-PAGE and then transferred to nitrocellulose membranes using a Bio-Rad *Trans*-Blot SD Semi-Dry Transfer Cell system. After incubation overnight at 4°C using a primary antibody, the blots were incubated with a secondary antibody (926-32211; LI-COR Biosciences, Lincoln, NE, United States). Then, an Odyssey infrared 740 imager (9120; LI-COR Biosciences) was used to scan the blots. H3K4me1, H3K4me2, H3K4me3, H3K9ac, H3K18me1, H3K23ac, H3K79me2, and H3K79me3 antibodies were obtained from EASYBIO (BE3281, BE3275, BE3224, BE3276, BE3291, BE3227, BE3301, and BE3302; Beijing, China). MYC and HA antibodies were purchased from Abmart (M20002 and M20003; Shanghai, China). FLAG antibodies were purchased from Bioregent Bio company (AB1027t; Beijing, China) and Abmart (M20008L; Shanghai, China). The Pgk1p polyclonal antibody was generated in rabbits.

### Oxygen Consumption Measurement

The Seahorse XF96 Extracellular Flux Analyzer (Seahorse Bioscience, Agilent Technologies) was used to detect respirations of WT and mutant yeast cells. The methods were similar as previous description (Bradley et al., 2020). 25 μL 50 μg/mL Poly-D-Lysine were added to each well of the Seahorse plate and incubated for 30 min at room temperature. Then the solution was removed and the plate was dried overnight. The Seahorse XF96 Sensor Cartridge was hydrated with 200 μL water overnight at 37°C, and the Seahorse XF Calibrant Solution was also kept at 37°C overnight. Before experiments, the water was replaced by 200 μL Seahorse XF Calibrant Solution and incubated 1 h at 37°C.

Yeast cells were cultured in YPD overnight at 30°C. On the day of measurement, cells were diluted to OD_600_ = 0.2. After 4 h culture, the exponential phase cells were harvested and resuspended in YPD, YPA, YPG (1% yeast extract, 2% peptone, and 2% glycerol), and YPE (1% yeast extract, 2% peptone, and 2% ethanol), respectively. 175 μL of each kind of cultures were added into the treated Seahorse plate that each well contained about 1 × 10^5^ cells. Then the plate was centrifuged for 3 min at 500 × *g* and incubated for 30 min at 30°C before loaded into the analyzer. The measure times of basal oxygen consumption rates were 2.5 min, 30 s, and 2.5 min. And 10 cycles were detected.

### Yeast Total RNA Extraction

Yeast total RNA extraction was accomplished using previously described methods (Schmitt et al., 1990). Briefly, corresponding sporulation samples were collected and mixed with 400 μL AE buffer (50 mM sodium acetate pH = 5.3 and 10 mM ethylene diamine tetraacetic acid), and 40 μL 10% SDS. After vibration 5 min using a vortex mixer (Scientific Industries, United States), 400 μL phenol was added and vibrated for another 5 min. Samples were next incubated for 4 min at 65°C and instantly cooled on ice. After centrifuged at 13,523 × *g* for 2 min, the supernatant was transferred to a new RNase free tube. The samples were then mixed with phenol and chloroform (1:1) and held at room temperature for 5 min, and the supernatant was again transferred to a new tube after centrifuging at 13,523 × *g* for 5 min. 2.5 × volume of ethanol and 40 μL 3M sodium acetate were added to the samples, which was finally centrifuged at 13,523 × *g* for 10 min to collect the RNA. 1 mL 80% ethanol was used to wash the RNA pellet and the supernatant was removed again after centrifuging at 13,523 × *g* for 10 min. Finally, the RNA pellet was dried, dissolved in 20 μL diethyl pyrocarbonate-treated water, and stored at −80°C.

### Quantitative Real-Time PCR

A PrimeScript RT Reagent Kit (RR037A, TaKaRa, Kusatsu, Japan) was used to synthesize complementary DNA (cDNA). Amplification was performed in a 10 μl reaction containing 5 μL 2 × EvaGreen mix (Master Mix-S; Applied Biological Materials, Richmond, Canada), 0.5 μL each primer (10 nmol/L), 2 μL sample cDNA, and 2.5 μL ddH2O. A Roche Light Cycler 480II System (Roche Diagnostics, Mannheim, Germany) was used to perform real-time PCR. The PCR program was initiated at 95°C for 10 min, followed by 40 cycles of denaturation for 5 s at 95°C, annealing for 30 s at 60°C, and elongation for 60 s at 72°C. Fluorescence signals were detected at 72°C in the time of the elongation step. Three biological replicates were taken for each sample and normalized to housekeeping gene *TAF10*. A Light Cycle 480 software 1.5.1 was used to analyzed the results. All primers used are shown in Supplementary Table 7.

### Statistical Analysis

All data were shown as the mean ± SD and statistic differences were calculated using a two-tailed Student’s *t*-test. The data were considered significant when the *P*-value was less than 0.05 (^∗^), 0.01 (^∗∗^), or 0.001 (^∗∗∗^).

## Results

### Histone H3 Acetylation and Methylation Are Dynamically Changed During Mouse Spermatogenesis

**FIGURE 1:**
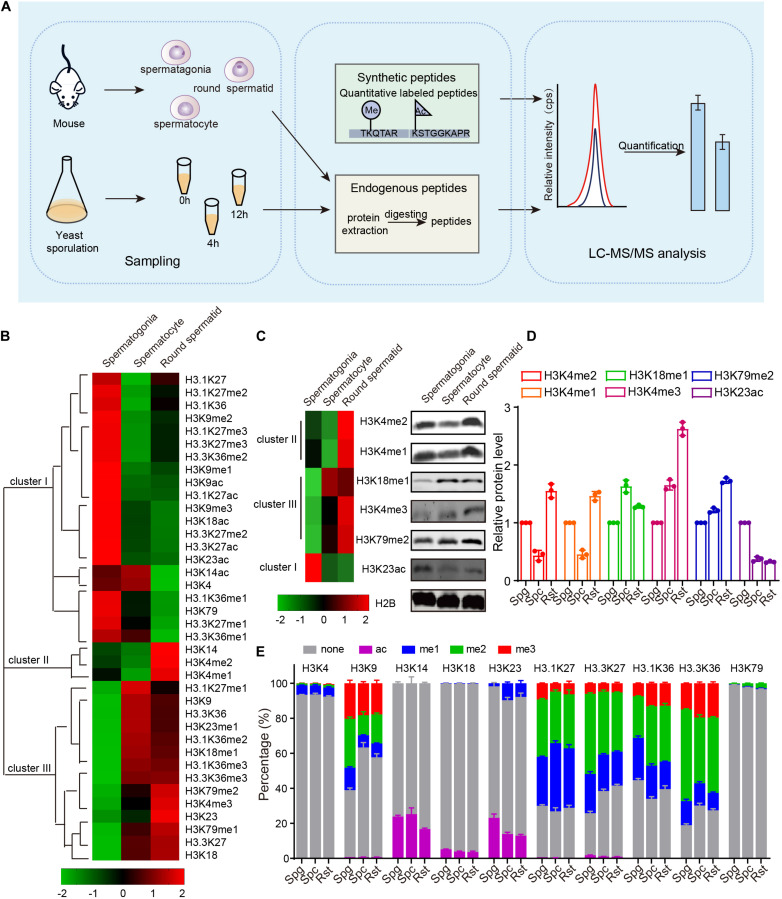
Dynamic changes of mouse histone H3 modifications during spermatogenesis. **(A)** Schematic of MRM-based LC-MS/MS. **(B)** Hierarchical clustering analysis of histone H3 modifications during mouse spermatogenesis. **(C)** Western blot verification of MS data. Protein samples for both western blots and MS were extracted from mouse cells using the same methods. **(D)** Quantitative of the histone modifications level in **C**. The error bars indicate ± SD (*n* = 3). Spg, spermatogonia; Spc, spermatocytes; Rst, round spermatids. **(E)** Relative abundance of histone modifications at histone H3 lysine sites for mouse MS samples. The error bars indicate ± SD (spermatogonia, *n* = 4; spermatocytes, *n* = 4; round spermatids, *n* = 3). Spg, spermatogonia; Spc, spermatocytes; Rst, round spermatids.

To assess the role of chromatin changes during gametogenesis, we detected the acetylation and methylation of H3 and its variants by using multiple reaction monitoring (MRM)-based LC-MS/MS of three crucial germ cell stages of spermatogenesis in mouse: spermatogonia, spermatocytes and round spermatids (Figure 1A). Proteins prepared from the separated cells were mixed with isotope-labeled histone peptides (Supplementary Table 3) and then analyzed using MRM-based LC-MS/MS. The concentration of the histones modifications during meiosis was measured by using isotope-labeled histone peptides as standards. Finally, we identified a total of 28 PTMs with 10 N-terminal lysine sites of acetylation, methylation in H3 and its variants, and found acetylation and methylation of H3 and its variants displayed dynamic change during spermatogenesis (Figure 1B and Supplementary Table 4).

These dynamically changed H3 modifications were clustered into three groups using Cluster 3.0 software (Figure 1B). Cluster I modifications showed an obvious decrease from spermatogonia to round spermatids, while cluster III modifications exhibited a gradual increase from spermatogonia to round spermatids (Figure 1B). Cluster II modifications showed decreases from spermatogonia to spermatocytes but increases in round spermatids (Figure 1B). The H3 modification trends in our data were similar to some previously reported, such as H3K4me1, H3K4me2, and H3K9me3 (Godmann et al., 2007; Iwamori et al., 2011), indicating our mass spec data could profile H3 acetylation and methylation during spermatogenesis quantitatively. To further confirm the data, we examined some H3 modification levels during spermatogenesis by using immunoblotting and found most of these H3 modifications were consistent with our MRM data (Figures 1C,D).

As MRM-based LC-MS/MS could determine the concentration of each histone modification, we attempted to quantify the precise percentage of H3 modifications at different lysine sites during spermatogenesis and found that modifications on different lysine sites in H3 or its variants were dramatically different from each other (Figure 1E and Supplementary Table 4). For example, rare modifications were found on H3K4, H3K18, and H3K79, whereas more than half the H3K9, H3.1K27, and H3.3K27 sites were modified by acetylation or methylation (Figure 1E). In these enriched modification sites, modifications were dramatically different between the three crucial stages of spermatogenesis (Figure 1E). We speculated that prevalent high percentage modifications might tend to have significant functions, like H3K9 methylation (Figure 1E). We also noticed that H3-variants modification were more prevalent than H3 modification (Figure 1E), indicating the H3 variants might play important roles in spermatogenesis. As the amount of histone were also dynamically changed during mouse spermatogenesis, the proportion of each kind of modifications on its own site might be different with the trend for the concentration of the histone modifications in Figure 1B. Taken together, our findings show MRM-based LC-MS/MS can profile H3 acetylation and methylation during spermatogenesis quantitatively, and H3 acetylation and methylation dynamically change during mouse spermatogenesis.

### PTMs of Histone H3 Dynamically Change During Yeast Sporulation

**FIGURE 2:**
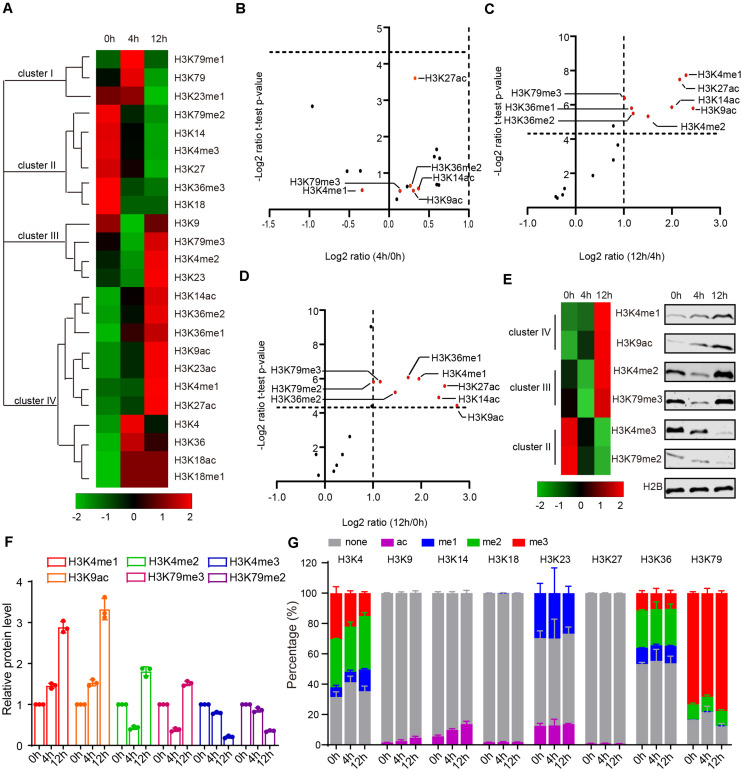
Dynamic changes of yeast histone H3 modifications during sporulation. **(A)** Hierarchical clustering analysis of histone H3 modifications during yeast sporulation. **(B–D)** Volcano Plot indicates upregulated and downregulated histone H3 modifications comparing 4 h/0 h, 12 h/4 h, and 12 h/0 h, respectively. In **(C,D)**, histone H3 modifications with a fold change >2 and a *P-*value < 0.05 are shown in red. **(E)** Western blot verification of MS data. Protein samples for both western blots and MS were extracted from yeast cells using the same methods. **(F)** Quantitative results of **E**. The error bars indicate ± SD (*n* = 3). **(G)** Relative abundance of histone modifications at histone H3 lysine sites for yeast MS samples.

Because we lack a direct way to manipulate histone modification sites in mice, it is difficult to directly assess the function of histone modification in mammals. Given yeast’s genetic operability and conservatism with mammals, budding yeast is a powerful model to study the functions of histone modifications in meiosis (Jin and Neiman, 2016; Owens et al., 2018). To explore the functional roles of H3 acetylation and methylation during meiosis, we examined levels of H3 acetylation and methylation by MRM-based LC-MS/MS in yeast after incubating with sporulation medium (SPM) at 0, 4, and 12 h (Figure 1A and Supplementary Table 5). As these three time points successively represent the early phase, meiosis process and spore formation in sporulation, they roughly correspond to the stages of spermatogonia, spermatocyte and round spermatids in mouse spermatogenesis (Cooper and Strich, 2011). Together, we identified a total of 16 PTMs with 8 N-terminal lysine sites of acetylation and methylation in H3 and found that, similar to the mouse results, acetylation and methylation of H3 displayed dynamic changes during yeast sporulation (Figure 2A and Supplementary Table 6).

During yeast sporulation, four types of H3 modifications were identified using Cluster 3.0 software (Figure 2A). Cluster I modifications showed a strong presence at 4 h, while cluster III modifications exhibited an obvious decrease from 0 to 4 h but an increase at 12 h (Figure 2A). Cluster II modifications exhibited a gradual decrease from 0 to 12 h (Figure 2A). In contrast, cluster IV modifications showed a gradual increase from 0 to 12 h (Figure 2A). We further compared these modifications at different yeast sporulation stages, and found H3K4me1, H3K9ac, H3K14ac, H3K27ac, and H3K36me1 showed obvious dynamic changes during yeast sporulation (Figures 2B–D). To further confirm our findings, we examined some H3 modification levels by immunoblotting during yeast sporulation and found most of these H3 modifications were similar to our MRM data (Figures 2E,F), indicating our methods could quantitatively profile H3 acetylation and methylation during yeast sporulation.

Next, we quantified the precise percentage of H3 modifications at different lysine sites during yeast sporulation. Similar to our MRM-based LC-MS/MS results in mice, some H3 lysine sites showed rare modification (e.g., H3K9, H3K14, H3K18, and H3K27), whereas other H3 lysine sites showed that more than 50% of the regions could be modified by acetylation or methylation (Figure 2G and Supplementary Table 6). At these enriched modified lysine sites, modification percentages, as well as specific H3 acetylation and methylation markings, showed dynamics changes during sporulation (Figure 2G and Supplementary Table 6).

To examine the conservation of H3 acetylation and methylation in mouse and yeast meiosis, we compared MRM-based LC-MS/MS results from mouse and yeast. Although some H3 modifications showed variable tendencies in mice and yeast, the dynamics of some H3 modifications during meiosis were conserved, such as H3K4me1, H3K4me2, and H3K18me1 (Supplementary Figure 1). In summary, H3 acetylation and methylation were dynamically changed during mouse and yeast meiosis, and the variation tendency of some H3 modifications during gametogenesis were conserved in both mice and yeast.

### Systematic Screening of H3 Lysine in Yeast

**FIGURE 3:**
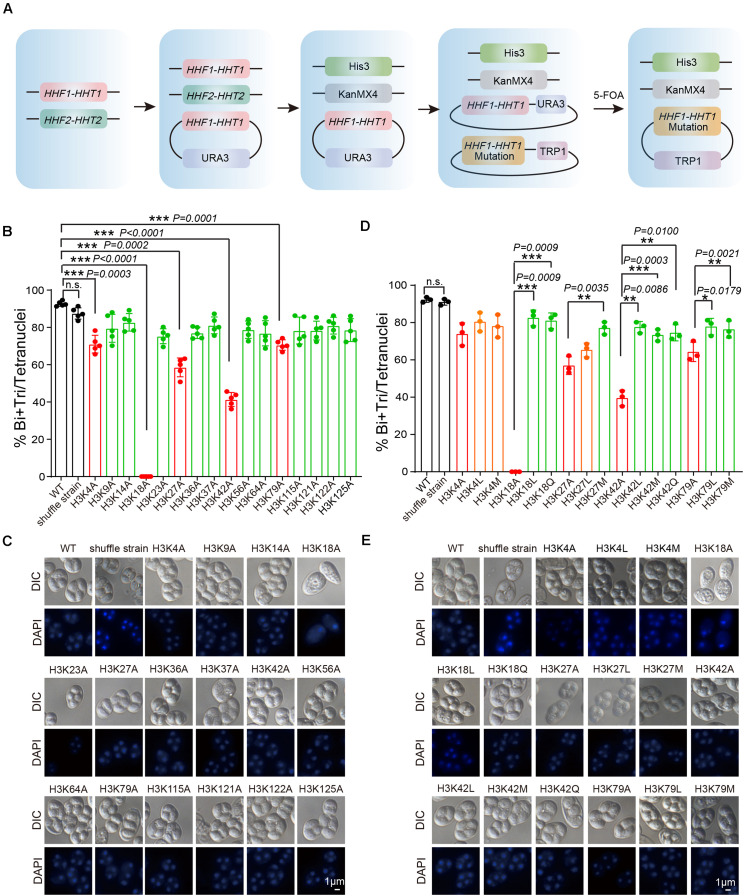
Systematic screen of H3 lysine modifications needed for sporulation. **(A)** H3 mutant strains were generated using an SK1 background. Endogenous histone H3 and H4 genes were knocked out, and the cell (LW1599) was transfected with a plasmid which had an Myc-*HHF1-HHT1*-HA and a *URA3* selection marker. Then, another plasmid with histone H3 point mutation was transfected and 5-FOA negative selection was used to replace the original plasmid. **(B)** Sporulation efficiency of histone H3 K to A mutant strains. The error bars indicate ± SD (*n* = 5). ***P < 0.001. n.s. indicates no significant. WT: LW0066; shuffle strain: LW1599; H3K4A: LW1600; H3K9A: LW1601; H3K14A: LW1602; H3K18A: LW1603; H3K23A: LW1604; H3K27A: LW1605; H3K36A: LW1606; H3K37A: LW1607; H3K42A: LW1608; H3K56A: LW1609; H3K64A: LW1610; H3K79A: LW1611; H3K115A: LW1612; H3K121A: LW1613; H3K122A: LW1614; H3K125A: LW1615. **(C)** Images of nuclei from cells in **B**. **(D)** Sporulation efficiency of mutant cells mimic different histone modifications. The error bars indicate ± SD (*n* = 3). *P < 0.05, **P < 0.01, ***P < 0.001. n.s. indicates no significant. WT: LW0066; shuffle strain: LW1599; H3K4A: LW1601; H3K4L: LW1616; H3K4M: LW1617; H3K18A: LW1603; H3K18L: LW1622; H3K18Q: LW1623; H3K27A: LW1605; H3K27L: LW1624; H3K27M: LW1625; H3K42A: LW1608; H3K42L: LW1626; H3K42M: LW1627; H3K42Q: LW1628; H3K79A: LW1611; H3K79L: LW1629; H3K79M: LW1630. **(E)** Images of nuclei from cells in **D**.

To further assess the functional role of H3 acetylation and methylation during meiosis, we performed a screen using systematic mutagenesis to substitute histone H3 lysine sites in yeast, based on a “histone shuffle” approach as previously described (Govin et al., 2010; Figure 3A). First, we deleted endogenous H3 and H4 genes (*HHF1*, *HHF2*, *HHT1*, and *HHT2*), and stabilized histone H3 and H4 protein levels by transforming a plasmid that contained *HHT1*, *HHF1*, and a *URA3* selectable marker (Figure 3A). Next, multiple H3 lysine mutations were included in a second plasmid (Lysine mutate to Alanine) included the WT promoter, making histone H3 protein expression similar to that of WT (Figure 3A). After the mutant plasmids were established, the original WT plasmid was removed using negative selection of *URA3* with 5-Fluoroorotic Acid (5-FOA) (Figure 3A and Supplementary Figure 2). Examination of the shuffle yeast strain showed that endogenous H3 proteins were absent, which could be replaced by the FLAG-tagged versions of histone (Supplementary Figure 2). Importantly, the sporulation efficiency of the shuffle strain was comparable to that of the WT strain (Figure 3B).

Next, we performed sporulation of these H3 mutant strains and found that the sporulation efficiency of H3K4A, H3K18A, H3K27A, H3K42A, and H3K79A mutants showed significant decreases compared with that of the WT and shuffle strain (Supplementary Figure 3 and Figures 3B,C), indicating the modifications on these sites might be very important to yeast sporulation. To further examine which modification types on these sites is important for yeast sporulation, we mutated Lysine (K) to Glutamine (Q) to mimic acetylation, to Leucine (L) to mimic mono-methylation, or to Methionine (M) to mimic di-methylation (Hyland et al., 2011; Supplementary Figure 4). We next determined the modification effects on sporulation efficiency. We found that H3K18Q and H3K18L could partially rescue impaired sporulation in H3K18A, indicating persistent H3K18ac and H3K18me were critical for yeast sporulation (Figures 3D,E). Similar results were observed for H3K27M, H3K42L, H3K42M, H3K42Q, H3K79L, and H3K79M, which suggest acetylation and methylation on H3K27, H3K42, and H3K79 might play important roles in yeast sporulation (Figures 3D,E). Given that the sporulation efficiency of H3K4L and H3K4M strains showed no significant differences compared with that of the H3K4A strain, H3K4me3 may be essential for proper meiosis progress, which is consistent with previous reported roles of H3K4me3 during meiosis (Adam et al., 2018). In addition, we also noticed that although H3K9A had no influence on yeast sporulation, H3K9M impaired sporulation progress (Supplementary Figure 5), indicating that persistent H3K9me2 might also influence yeast sporulation. Together, our results indicate that acetylation and methylation on H3K18, H3K27, H3K42, and H3K79 are very important to meiosis in yeast.

### H3K18ac Facilitates Sporulation Initiation Through Respiration

**FIGURE 4:**
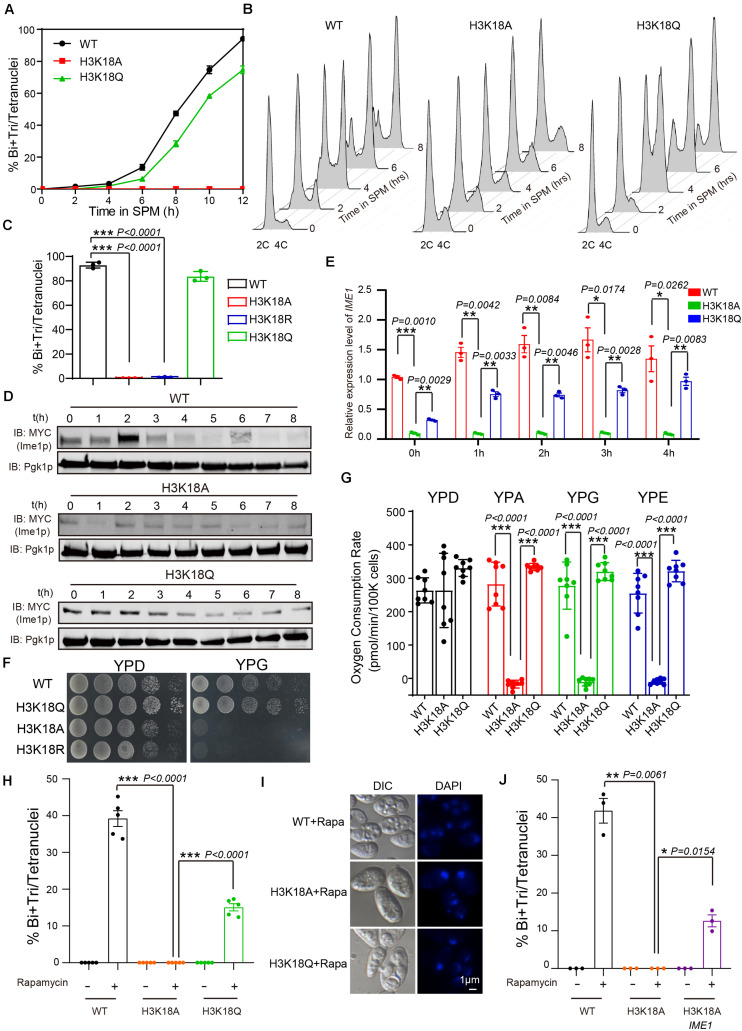
Persistent H3K18ac state is sufficient to induce yeast sporulation. **(A)** Sporulation curve of WT, H3K18A and H3K18Q mutant strains in SPM from 0 h to 12 h. 300 μL samples were fixed with 1 mL 70% ethanol for at least 1 h and then stained with DAPI to calculate sporulation rates. The error bars indicate ± SD (*n* = 3). **(B)** Flow cytometry of the WT, H3K18A, and H3K18Q mutant strains. Samples were prepared at different times after being cultured in SPM to detect premeiotic DNA replication (2C-4C transition). Premeiotic DNA replication was blocked in the H3K18A mutant strain, whereas the H3K18Q mutant strain showed active premeiotic DNA replication. **(C)** Sporulation efficiencies of WT, H3K18A, H3K18R, and H3K18Q. The error bars indicate ± SD (*n* = 3). ***P < 0.001. H3K18R: LW1643. **(D)** Western blot of Ime1p expression in WT, H3K18A, and H3K18Q mutant strains during sporulation. *IME1* in all three strains was tagged with 9 × Myc. Pgk1p was used as a loading control. WT-*IME1*-9MYC: LW1631; H3K18A-*IME1*-9MYC: LW1632; H3K18Q-*IME1*-9MYC: LW1633. **(E)** Quantitative PCR analyzed *IME1* expression in WT, H3K18A, and H3K18Q mutant strains. Cells were harvested at the indicated times and then total RNA was extracted and reverse transcribed. *IME1* mRNA levels in the three strains were measured by quantitative PCR and normalized to the levels of the house keeping gene *TAF 10*. The error bars indicate ± SD (*n* = 3). *P < 0.05, **P < 0.01, ***P < 0.001. **(F)** Detection of respiration function of H3K18 mutant strains. Left panel: cells from WT and mutant strains were spotted on YPD plates as serial 10-fold dilutions. Right panel: cells from WT and mutant strains were also spotted on YPG plates (a medium using non-fermentable carbon source glycerol). **(G)** Oxygen consumption rates (OCR) of WT and mutant cells in fermentative and non-fermentative carbon source medium. Lack of H3K18ac showed respiration defect in YPA (yeast extract, peptone, potassium acetate), YPG (yeast extract, peptone, glycerol) and YPE medium (yeast extract, peptone, ethanol). The error bars indicate ± SD (*n* = 8). ***P < 0.001. **(H)** H3K18 acetylation played important roles in sporulation induced by rapamycin in YPD medium. The indicated cells were cultured in YPD for 24 h at 30°C and then induced by rapamycin or mock-induced by methanol. Sporulation efficiencies were analyzed at 36 h after treatment. The error bars indicate ± SD (*n* = 5). ***P < 0.001. **(I)** Images of nuclei from cells in **H**. **(J)** Overexpression of Ime1p in the H3K18A mutant strain could partly rescue sporulation induced by rapamycin in YPD medium. The indicated cells were cultured in YPD for 24 h at 30°C, then induced by rapamycin or mock-induced by methanol. Sporulation efficiencies were analyzed at 36 h after treatment. The error bars indicate ± SD (*n* = 3). *P < 0.05, **P < 0.01. H3K18A Ime1p: LW1640.

Given that H3K18A absolutely blocked the sporulation progress (Figures 3D, 4A) and that H3K18ac showed a higher proportion of K18 modification in H3 during yeast sporulation (Figure 2G and Supplementary Table 6), we selected H3K18ac for further analyze. To examine which stage was influenced during H3K18A mutant strain sporulation, we first used flow cytometry analysis to detect premeiotic DNA replication in the H3K18A mutant strain. In WT cells, premeiotic DNA replication was initiated at 2 h and finished at 4 h when cells were cultured in sporulation medium (Figure 4B). Contrarily, with the H3K18A mutant, premeiotic DNA replication did not even initiate at 10 h after the cells were cultured in SPM (Figure 4B). Thus, H3K18ac might be required for premeiotic DNA replication. To further confirm our findings, we generated H3K18R and H3K18Q strains which keeps the positive charge while mimic a non-acetylated state or a persistent H3K18ac state, respectively. We found that the sporulation of H3K18R mutant was blocked which is similar to that of the H3K18A mutant (Figure 4C), while the H3K18Q could partially rescue the sporulation progress (Figures 4A,C). Furthermore, the H3K18Q was sufficient to initiate premeiotic DNA replication (Figure 4B). Thus, our findings suggest that the absence of H3K18ac might perturb yeast sporulation by blocking premeiotic DNA replication.

As Ime1p serves as a master regulator to initiate yeast premeiotic DNA replication (Kassir et al., 1988; Vershon and Pierce, 2000), we evaluated the expression of Ime1p in H3K18A mutants. We found Ime1p expression was dramatically decreased in H3K18A mutants compared with that in WT strain, and the mimic H3K18ac in the H3K18Q mutant could partially rescue Ime1p protein levels (Figure 4D). Given that histone modifications commonly regulate gene expression (Fukuda et al., 2006; Rintisch et al., 2014), we speculated that H3K18ac might modulate Ime1p protein levels by regulating *IME1* expression. To test our speculation, we examined mRNA levels of *IME1* in WT, H3K18A and H3K18Q strains using quantitative real-time PCR and found that levels of *IME1* mRNA in WT, H3K18A, and H3K18Q strains correlated with their protein levels (Figure 4E), suggesting H3K18ac might modulate meiosis initiation by regulating *IME1* expression.

Many intrinsic and extrinsic signals have been reported to modulate the expression of *IME1* (Jaiswal et al., 2017), and among them, respiration has been shown to be required for *IME1* expression during meiosis (Jambhekar and Amon, 2008; Zhao et al., 2018). Therefore, we speculated that H3K18ac might regulate *IME1* expression via respiration. To test our hypothesis, we cultured WT and H3K18A mutant cells on YPG medium, which contains a non-fermentable carbon source that respiration-deficient cells cannot use. Compared to the WT strain, the H3K18A mutant strain displayed obvious growth defects (Figure 4F), whereas the H3K18Q mutant strain did not (Figure 4F). Moreover, the H3K18R mutant strain displayed obvious growth defects in YPG (Figure 4F), which further supports H3K18A effect can be attributed to acetylation. Therefore, H3K18ac might participate in respiration. To further confirm it, we measured the respiratory capacity of WT and mutant strains in different medium by detecting the oxygen consumption rate via Seahorse XF96 Extracellular Flux Analyzer. We found that the WT, H3K18A, and H3K18Q strains respired normally in YPD medium, when cultured in YPA, YPG, or YPE medium, only H3K18A strain displayed respiration defect, but not the WT or H3K18Q strains (Figure 4G). As respiration could also provide energy by using a non-fermentable carbon source during yeast sporulation, we sought to distinguish the effect of H3K18ac on energy supplement and *IME1* expression. To investigate, we induced the sporulation of WT and H3K18A mutant strains with rapamycin in YPD, in which glucose could supply an energy source (Zheng and Schreiber, 1997). We found the H3K18A mutant strain still failed to undergo sporulation compared with the WT strain, whereas the H3K18Q mutant strain could overcome this defect (Figures 4H,I). Our findings suggest H3K18ac might regulate meiosis initiation through respiration by modulating *IME1* expression, but not energy supplementation. To further confirm our results, in the H3K18A mutant strain, we overexpressed *IME1* under the *CUP1* promoter and found *IME1* overexpression could partially rescue the H3K18A sporulation defects (Figure 4J). Thus, H3K18ac might regulate *IME1* expression through respiration.

### H3K18ac Regulates Ime1p Levels Through the Rim101p-Smp1p Pathway

**FIGURE 5:**
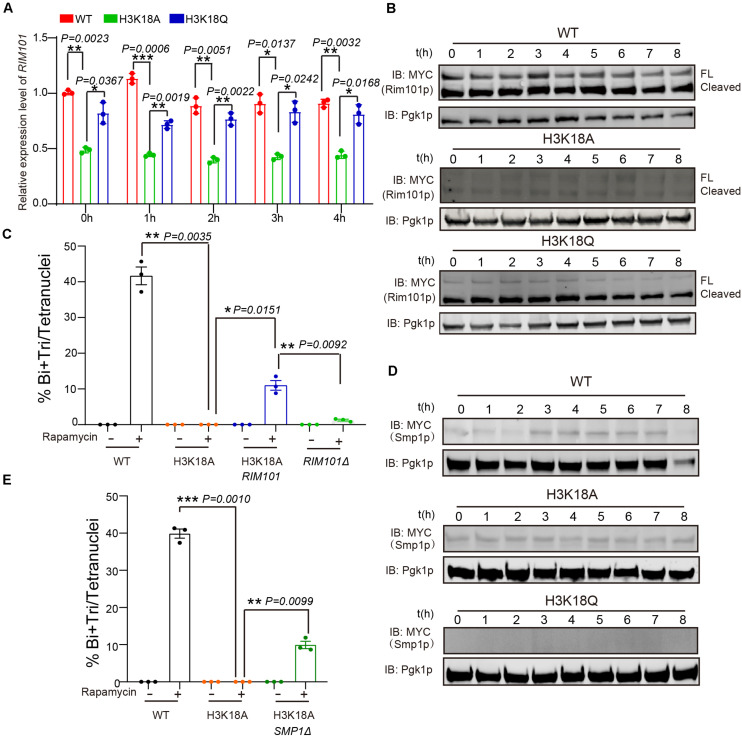
H3K18ac influences meiosis initiation by regulating *RIM101* and *SMP1* expression. **(A)** Quantitative PCR analyzed *RIM101* expression in WT, H3K18A and H3K18Q mutant strains. Cells were harvested at the indicated times and then total RNA was extracted and reverse transcribed. *RIM101* mRNA levels in the three strains were measured by quantitative PCR and normalized to the levels of the house keeping gene *TAF10*. The error bars indicate ± SD (*n* = 3). *P < 0.05, **P < 0.01, ***P < 0.001. **(B)** Western blot of Rim101p expression in WT, H3K18A, and H3K18Q mutant strains during sporulation. *RIM101* in all three strains was tagged with 9 × Myc. Pgk1p was used as a loading control. WT-9MYC-*RIM101*: LW1634; H3K18A-9MYC-*RIM101*: LW1635; H3K18Q-9MYC-*RIM101*: LW1636. **(C)** Overexpression of *RIM101* in H3K18A mutant strain could partly rescue the sporulation induced by rapamycin in YPD medium. Indicated cells were cultured in YPD for 24 h at 30°C, then induced by rapamycin or methanol as control. Sporulation efficiency were analyzed at 36 h after treatment. Error bar indicate ± SD (*n* = 3). *P < 0.05, **P < 0.01. H3K18A Rim101p: LW1641; *RIM101Δ*: LW1560. **(D)** Western blot of Smp1p expression in WT, H3K18A and H3K18Q mutant strains during sporulation. *SMP1* in all three strains was tagged with 9 × Myc. Pgk1p was used as a loading control. WT-*SMP1*-9MYC: LW1637; H3K18A-*SMP1*-9MYC: LW1638; H3K18Q-*SMP1*-9MYC: LW1639. **(E)** Delete *SMP1* in H3K18A mutant strain could partly rescue the sporulation induced by rapamycin in YPD medium. Indicated cells were cultured in YPD for 24 h at 30°C, then induced by rapamycin or methanol as control. Sporulation efficiency were analyzed at 36 h after treatment. Error bar indicate ± SD (*n* = 3). **P < 0.01, ***P < 0.001. H3K18A *SMP1Δ*: LW1642.

Previously, we found respiration could promote *RIM101* expression to inhibit Smp1p, which further activates *IME1* expression to facilitate meiosis initiation (Zhao et al., 2018). Therefore, we speculated that H3K18ac might modulate respiration and initiate meiosis through the Rim101p-Smp1p-Ime1p pathway. To test this hypothesis, we first measured *RIM101* mRNA levels in WT, H3K18A, and H3K18Q mutant strains, and found that the *RIM101* level in H3K18A strain was significantly decreased compared with that of the WT and H3K18Q strains (Figure 5A). To further confirm it, we then detected the Rim101p levels in WT, H3K18A, and H3K18Q mutant strains during sporulation, and found that only the Rim101p in H3K18A mutant strain was dramatically downregulated (Figure 5B). Furthermore, the H3K18Q mutant strain showed recovered Rim101p protein levels (Figure 5B), indicating H3K18ac might regulate Rim101p expression through respiration. To further confirm this idea, in the H3K18A mutant strain, we overexpressed *RIM101* under the *CUP1* promoter and found *RIM101* overexpression could partially rescue sporulation defects in H3K18A mutant strain (Figure 5C). Thus, H3K18ac might regulate meiosis initiation through modulating Rim101p expression.

**FIGURE 6:**
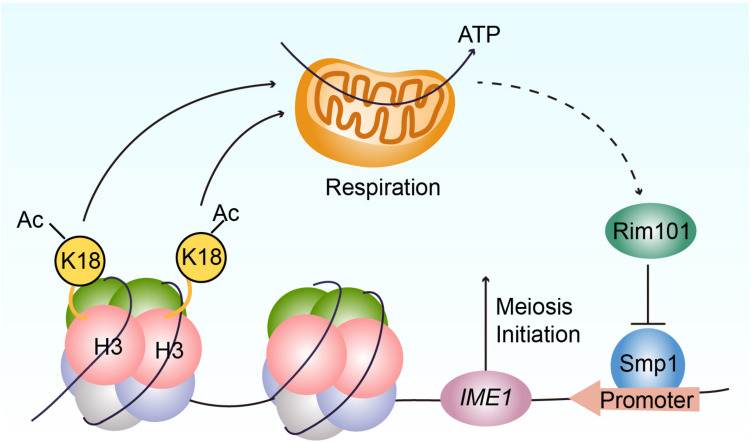
Proposed model for the functional role of H3K18ac in meiosis initiation. H3K18ac influences the respiration to promote *RIM101* expression that down-regulates *SMP1* a repressor of *IME1* transcription, induces *IME1* expression, and finally leads to meiosis initiation.

Given that Rim101p could activate *IME1* expression by down-regulating Smp1p (Zhao et al., 2018), we further examined the protein levels of Smp1p in WT, H3K18A, and H3K18Q mutant strains. We found Smp1p dramatically accumulated at the early stage of sporulation in the H3K18A mutant strain compared with the WT strain (Figure 5D), and the H3K18Q mutant strain showed recovered Smp1p protein levels (Figure 5D). In addition, the disruption of *SMP1* in the H3K18A mutant strain partially rescued sporulation defects (Figure 5E), suggesting H3K18ac might downregulate Smp1p through respiration. Thus, H3K18ac might modulate respiration and regulate meiosis initiation through the Rim101p-Smp1p-Ime1p pathway (Figure 6).

## Discussion

Histone modifications have been reported to be involved in many aspects of meiosis, including the generation of programmed DNA double-strand breaks (DSBs), homologous recombination, meiotic sex chromosome inactivation (MSCI) and crossover formation (Bannister and Kouzarides, 2011; Tan et al., 2011; Tessarz and Kouzarides, 2014). Although an appreciable number of studies have uncovered many histone modifications during meiosis, majority of the studies were descriptive and correlative. Our current study sought to elucidate how histone modifications directly or indirectly modulate meiosis. Quantitative analysis of prevalent histone proteins and their post-translational modifications (PTM) provided insight into understanding the mechanistic relationships between histone modifications and meiosis.

Based on our study, we speculated that highly prevalent modifications might tend to have significant functions, such as the H3K9 methylation that could be identified on more than half of the K9 sites in H3 during mouse spermatogenesis (Figure 1E). Indeed, impaired methylation of H3K9 (me1/3) led to abnormal meiosis homologous recombination, which could result in spermatogenesis failure (Tachibana et al., 2001). We also detected a high proportion of modifications on H3 variants (Figure 1E), suggesting important roles of PTMs during spermatogenesis. Indeed, some studies have revealed that H3 variants are essential for spermatogenesis (Jambhekar and Amon, 2008; Yuen et al., 2014), supported by findings that *H3f3b*-null male mice are complete infertile and show disrupted spermatogenesis-related genes expression in germ cells (Yuen et al., 2014). However, few works focused on the functional roles of H3 variants modifications, which need further investigation.

In the majority of eukaryotes, respiration is an important contributor of adenosine triphosphate (ATP), which supports a series of physiological function (Friedman and Nunnari, 2014). In budding yeast, a non-fermentable carbon source is critical for meiosis initiation (Zaman et al., 2008) and requires mitochondrial respiration to be utilized by the cell (Treinin and Simchen, 1993). In essence, the components of the mitochondrial respiratory chain are necessary for yeast sporulation (Treinin and Simchen, 1993; Zhao et al., 2018). In a previous study, we found respiration could activate *IME1* expression through the Rim101p-Smp1p pathway (Zhao et al., 2018). Because Ime1p is a master transcription regulator of yeast meiosis that activates early meiotic gene expression (van Werven and Amon, 2011), respiration could further promote meiosis initiation (Smith et al., 1990; Benjamin et al., 2003). Our current study finds that H3K18ac could modulate respiration to activate Rim101p expression, further downregulating a negative regulator of *IME1* transcription, Smp1p, to initiate meiosis (Figure 6).

According to our findings, H3K18ac plays important roles in meiosis initiation and histone acetylation is regulated by histone acetyl-transferase and deacetylase. Therefore, it is important to uncover how H3K18ac is regulated by enzymes during meiosis initiation. *SIRTUIN 7* (*SIRT7*) is known to be responsible for H3K18 deacetylation in mammals (Barber et al., 2012) and *SIR2* is the *SIRT7* homology in yeast (Paredes et al., 2018). SIRT7 methylated at R388 has been reported to lose its deacetylation activity on H3K18, and hyperacetylated H3K18 on SIRT7-target gene promoter has been reported to initiate mitochondria synthesis and maintain mitochondria respiration (Yan et al., 2018). Furthermore, a lack of Sir2p distinctly enhanced gluconeogenesis and respiration in yeast (Orlandi et al., 2017). Gcn5p-Ada2p-Ada3p was the catalytic subunit of the Ada2-Gcn5-Ada3 transcription activator (ADA) and Spt-Ada-Gcn5-acetyltransferase (SAGA) complexes, which showed acetylation activity on H3K18 (Cieniewicz et al., 2014), and Gcn5p was reported to play a central role in yeast meiosis initiation through histone H3 acetylation (Burgess et al., 1999). Given that H3K18ac was dynamically changed during meiosis in both mice and yeast (Figures 1, 2) albeit their trends are different from each other, more efforts should be made to understand the functional role of H3K18ac on spermatogenesis.

## Data Availability Statement

The data presented in the study are deposited in the PeptideAtlas repository, accession number (PASS01662).

## Ethics Statement

The animal study was reviewed and approved by the Animal Research Panel of the Committee on Research Practice of the Institute of Zoology, Chinese Academy of Sciences (#08-133).

## Author Contributions

WL, HY, and CL conceived and designed the experiments. JS, YM, and HH performed most of the experiments. YL performed some of the experiments. HY performed LC-MS/MS. JS, YM, CL, and WL wrote the article. All authors contributed to the article and approved the submitted version.

## Conflict of Interest

The authors declare that the research was conducted in the absence of any commercial or financial relationships that could be construed as a potential conflict of interest.

## Supplementary Material

Supplementary Figure 1 The comparison of histone modifications which show similar dynamic trends in mouse spermatogenesis and yeast sporulation. H3K4me1, H3K4me2, and H3K18me1 showed similar changes in both mouse and yeast during meiosis.

Supplementary Figure 2 Western blot verification of the construction of histone H3 K to A mutant strains. **(A)** The K to A mutant histone H3 could be expressed. Pgk1p was used as a loading control. **(B)** The original histone H3 could not be detected. 5-FOA was used to remove the plasmid expressing wild-type histone H3. Pgk1p was used as a loading control.

Supplementary Figure 3 Less zoomed-in versions of the sporulation images about the representative sporulation defect strains in Figure 3B.

Supplementary Figure 4 Western blot verification of the generated of histone H3 mimic strains. **(A–G)** The modification mimic histone H3 could be expressed and the original wild-type histone H3 plasmid was removed using negative section with 5-FOA. Pgk1p was used as a loading control.

Supplementary Figure 5 Modifications mimic strains to rescue the sporulation rate of H3K9A and H3K14A mutant strains. **(A)** Sporulation efficiencies of mutant cells mimic different histone modifications. The error bars indicate ± SD (*n* = 3). **P* < 0.05, ***P* < 0.01. WT: LW0066; H3K9A: LW1601; H3K9L: LW1618; H3K9M: LW1619; H3K9Q: LW1620; H3K14A: LW1602; H3K14Q: LW1621. **(B)** Images of nuclei of cells in **A**.
