# Supplementary figures and images for: Dynamic Histone H3 Modifications Regulate Meiosis Initiation via Respiration
Source: Front Cell Dev Biol. 2021 Apr 1;9:646214. doi: 10.3389/fcell.2021.646214 (PMC8047140; doi:10.3389/fcell.2021.646214)

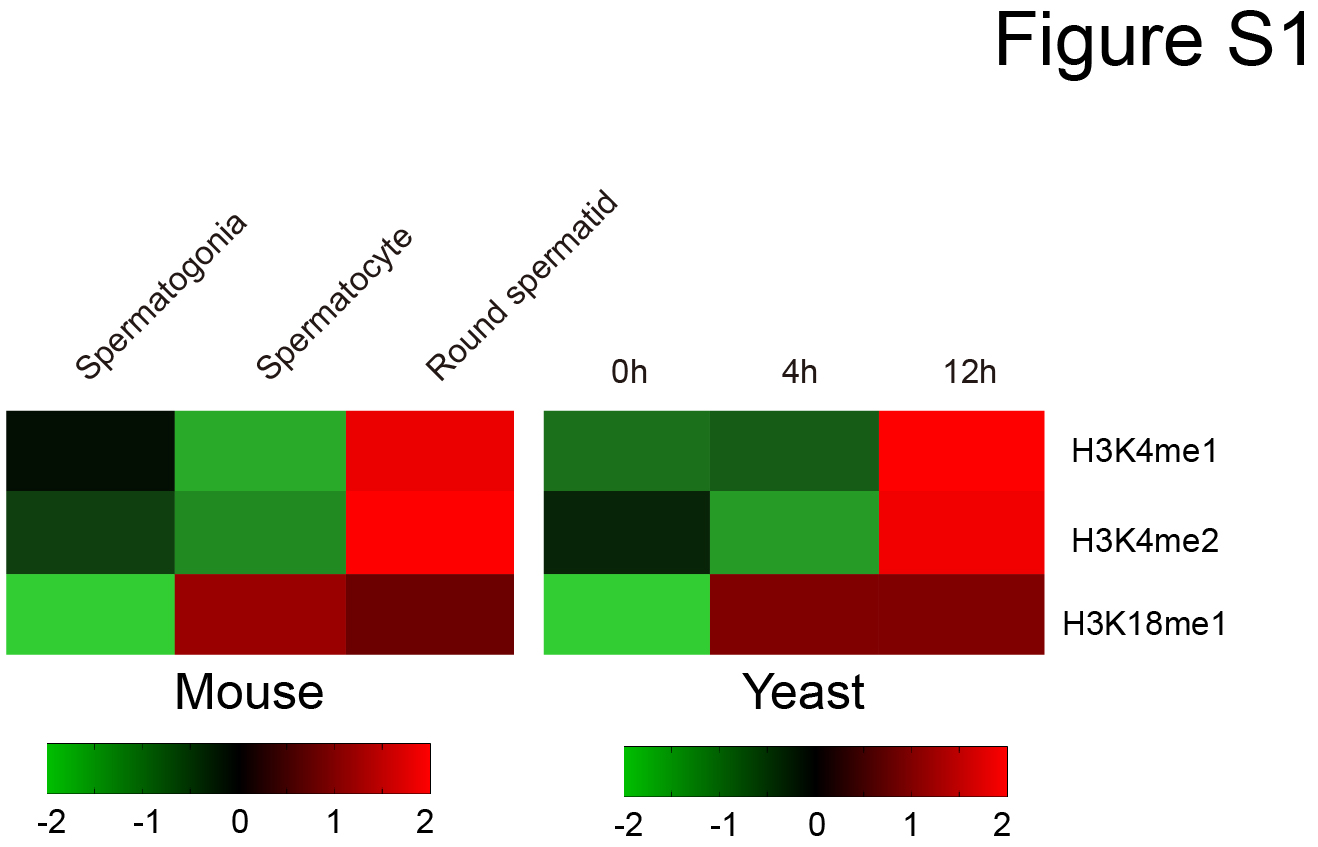

Supplement: Supplementary Figure 1 — The comparison of histone modifications which show similar dynamic trends in mouse spermatogenesis and yeast sporulation. H3K4me1, H3K4me2, and H3K18me1 showed similar changes in both mouse and yeast during meiosis. [file Image_1.JPEG]

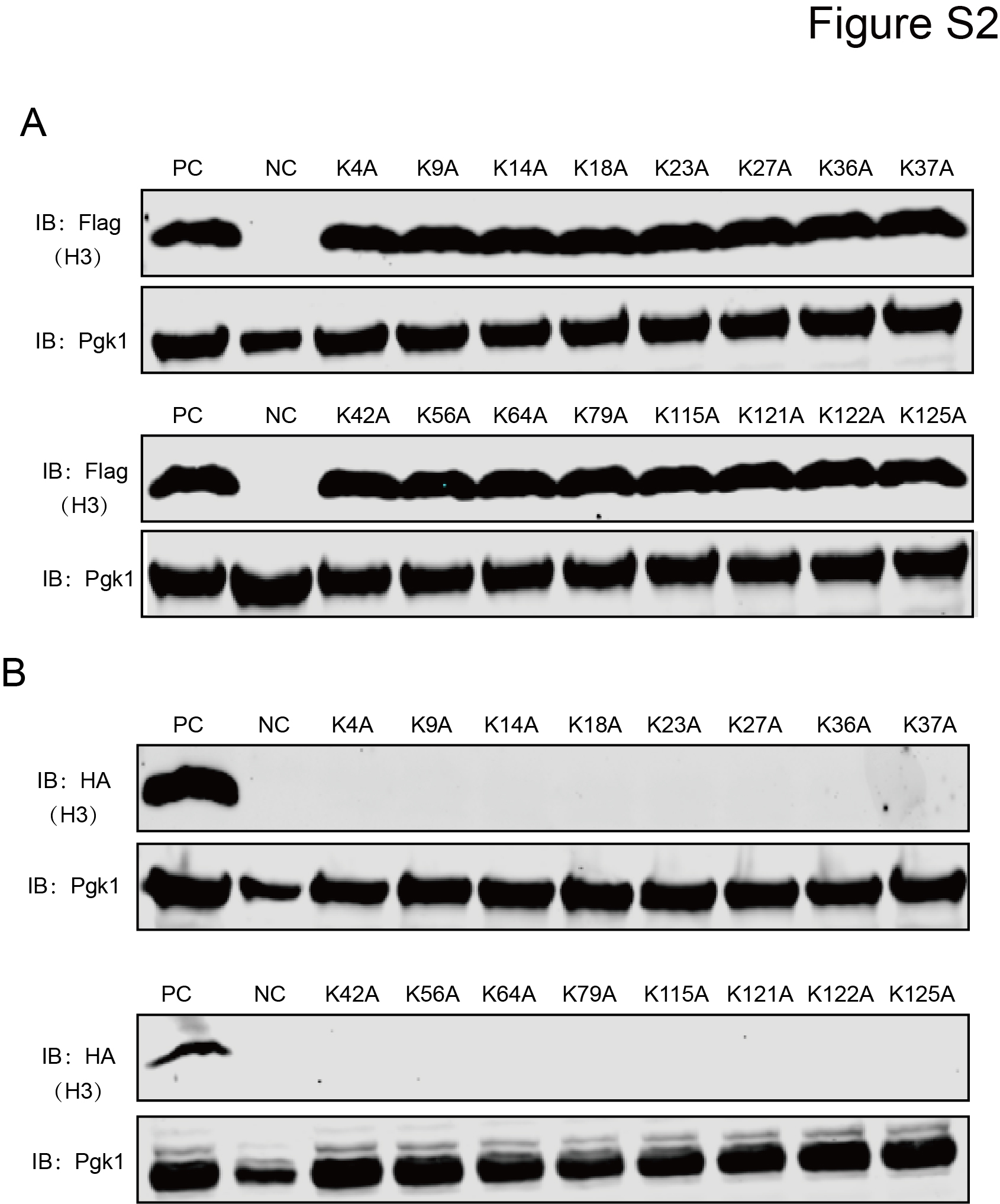

Supplement: Supplementary Figure 2 — Western blot verification of the construction of histone H3 K to A mutant strains. (A) The K to A mutant histone H3 could be expressed. Pgk1p was used as a loading control. (B) The original histone H3 could not be detected. 5-FOA was used to remove the plasmid expressing wild-type histone H3. Pgk1p was used as a loading control. [file Image_2.JPEG]

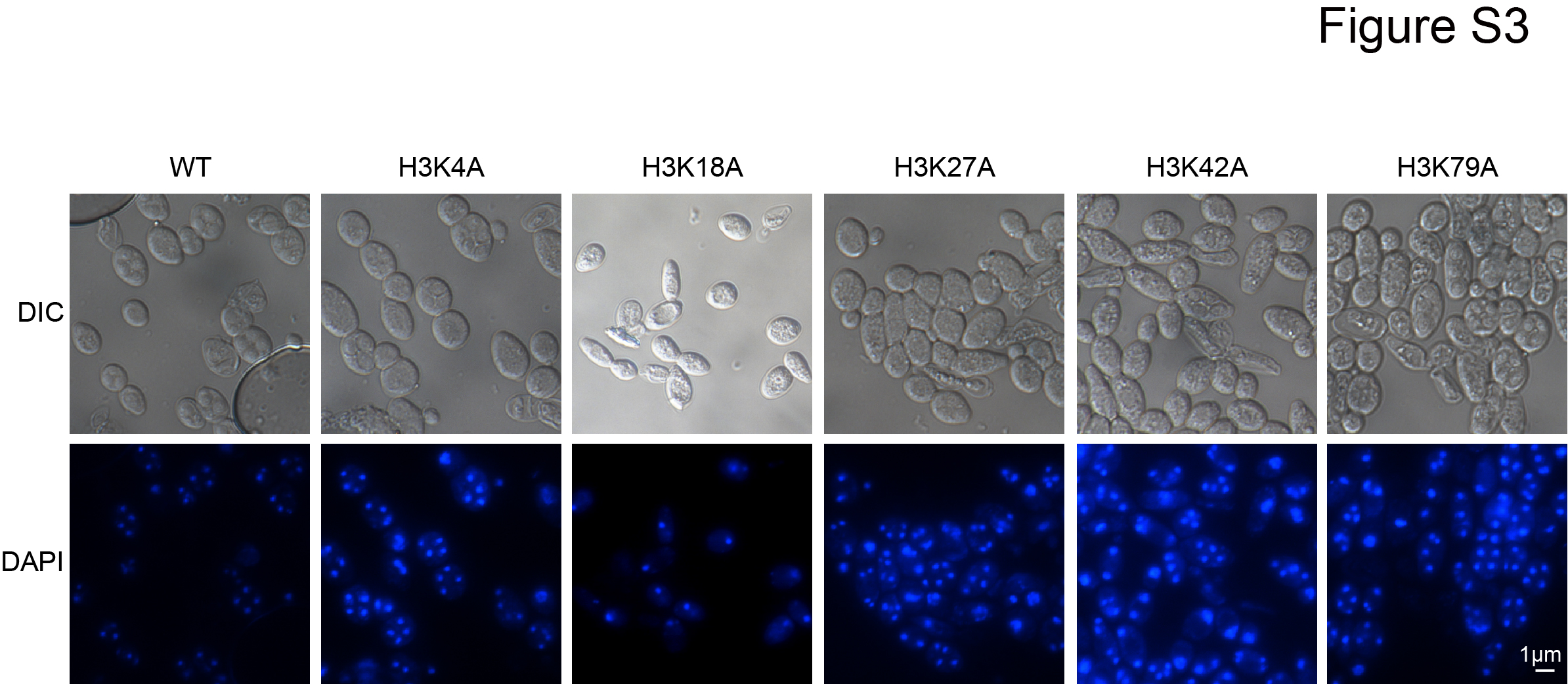

Supplement: Supplementary Figure 3 — Less zoomed-in versions of the sporulation images about the representative sporulation defect strains in Figure 3B. [file Image_3.JPEG]

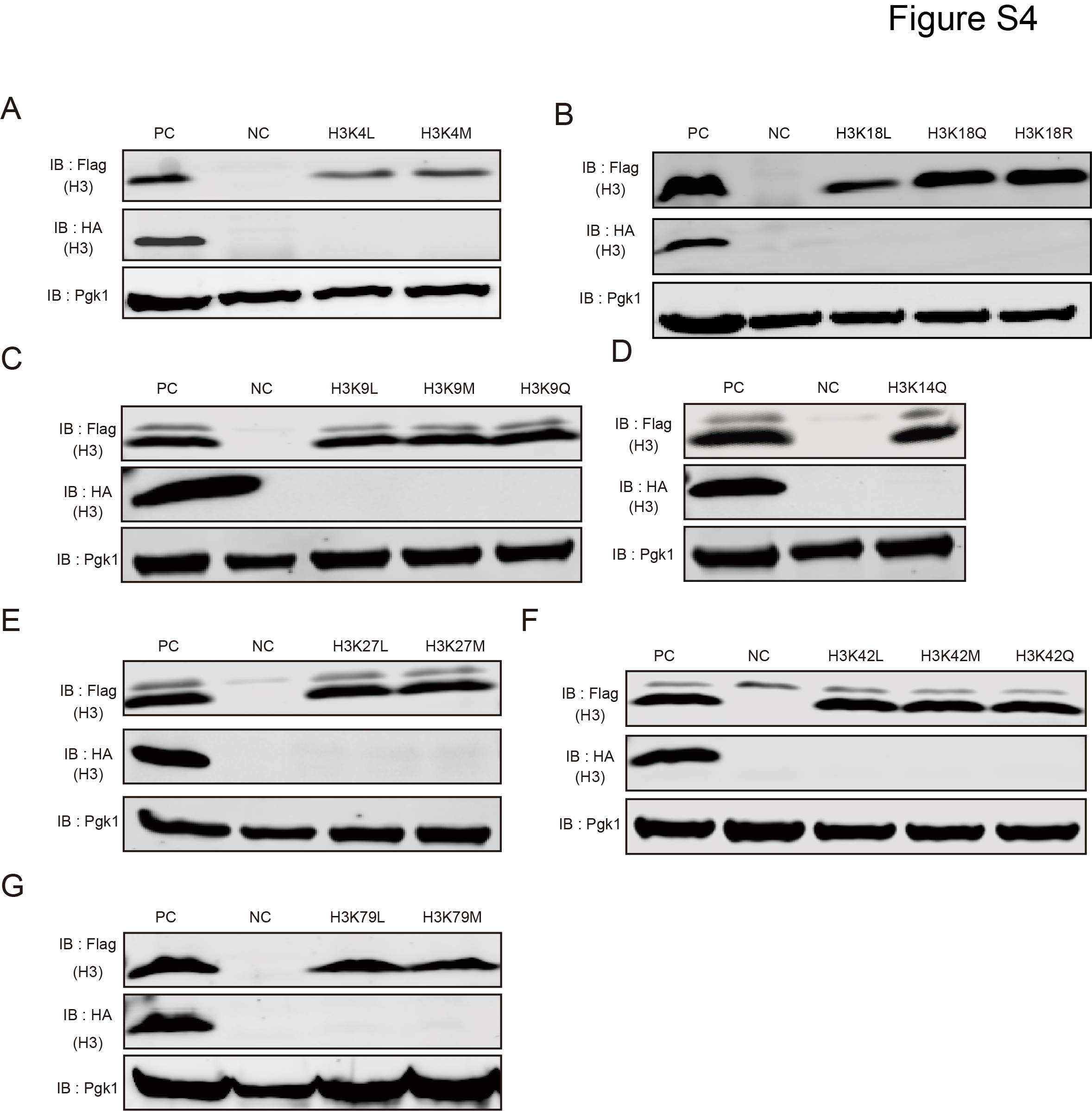

Supplement: Supplementary Figure 4 — Western blot verification of the generated of histone H3 mimic strains. (A–G) The modification mimic histone H3 could be expressed and the original wild-type histone H3 plasmid was removed using negative section with 5-FOA. Pgk1p was used as a loading control. [file Image_4.JPEG]

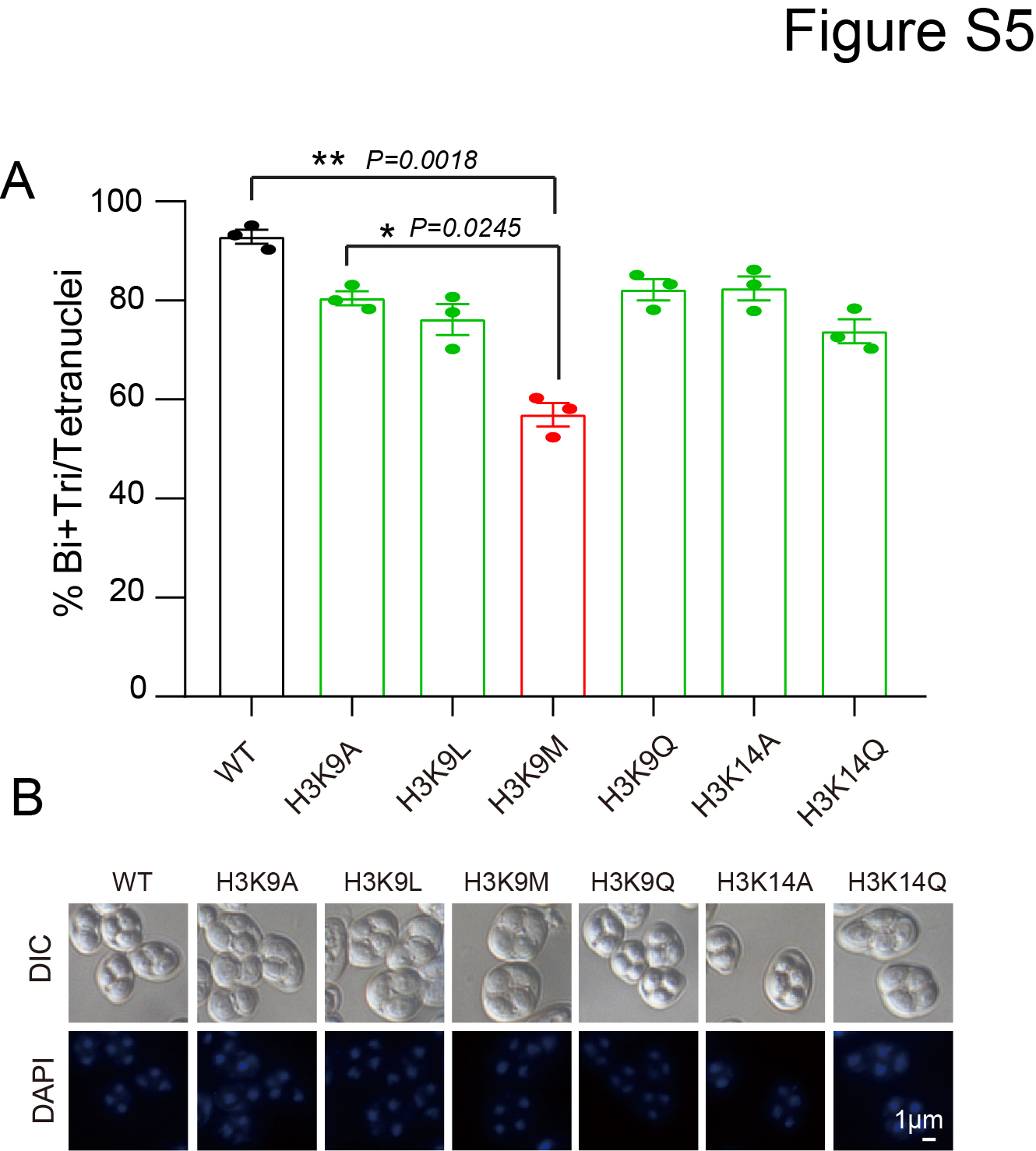

Supplement: Supplementary Figure 5 — Modifications mimic strains to rescue the sporulation rate of H3K9A and H3K14A mutant strains. (A) Sporulation efficiencies of mutant cells mimic different histone modifications. The error bars indicate ± SD (n = 3). *P < 0.05, **P < 0.01. WT: LW0066; H3K9A: LW1601; H3K9L: LW1618; H3K9M: LW1619; H3K9Q: LW1620; H3K14A: LW1602; H3K14Q: LW1621. (B) Images of nuclei of cells in A. [file Image_5.JPEG]
